# Ornithine Transcarbamylase – From Structure to Metabolism: An Update

**DOI:** 10.3389/fphys.2021.748249

**Published:** 2021-10-01

**Authors:** Morgane Couchet, Charlotte Breuillard, Christelle Corne, John Rendu, Béatrice Morio, Uwe Schlattner, Christophe Moinard

**Affiliations:** ^1^Université Grenoble Alpes, Inserm U1055, Laboratory of Fundamental and Applied Bioenergetics, Grenoble, France; ^2^Centre Hospitalier Université Grenoble Alpes, Grenoble, France; ^3^CarMeN Laboratory, INSERM U1060, INRAE U1397, Lyon, France; ^4^Institut Universitaire de France, Paris, France

**Keywords:** OTC deficiency, liver, intestine, NASH, diabetes, citrulline

## Abstract

Ornithine transcarbamylase (OTC; EC 2.1.3.3) is a ubiquitous enzyme found in almost all organisms, including vertebrates, microorganisms, and plants. Anabolic, mostly trimeric OTCs catalyze the production of L-citrulline from L-ornithine which is a part of the urea cycle. In eukaryotes, such OTC localizes to the mitochondrial matrix, partially bound to the mitochondrial inner membrane and part of channeling multi-enzyme assemblies. In mammals, mainly two organs express OTC: the liver, where it is an integral part of the urea cycle, and the intestine, where it synthesizes citrulline for export and plays a major role in amino acid homeostasis, particularly of L-glutamine and L-arginine. Here, we give an overview on OTC genes and proteins, their tissue distribution, regulation, and physiological function, emphasizing the importance of OTC and urea cycle enzymes for metabolic regulation in human health and disease. Finally, we summarize the current knowledge of OTC deficiency, a rare X-linked human genetic disorder, and its emerging role in various chronic pathologies.

## Introduction

Ornithine transcarbamylase (OTC, EC 2.1.3.3; also called ornithine carbamoyltransferase, OCT) is an evolutionary ancient enzyme, present in most organisms from bacteria to plants and vertebrates, and mostly encoded by a single nuclear gene. It is part of the transcarbamylase protein family (Shi et al., 2015).

**Figure 1:**
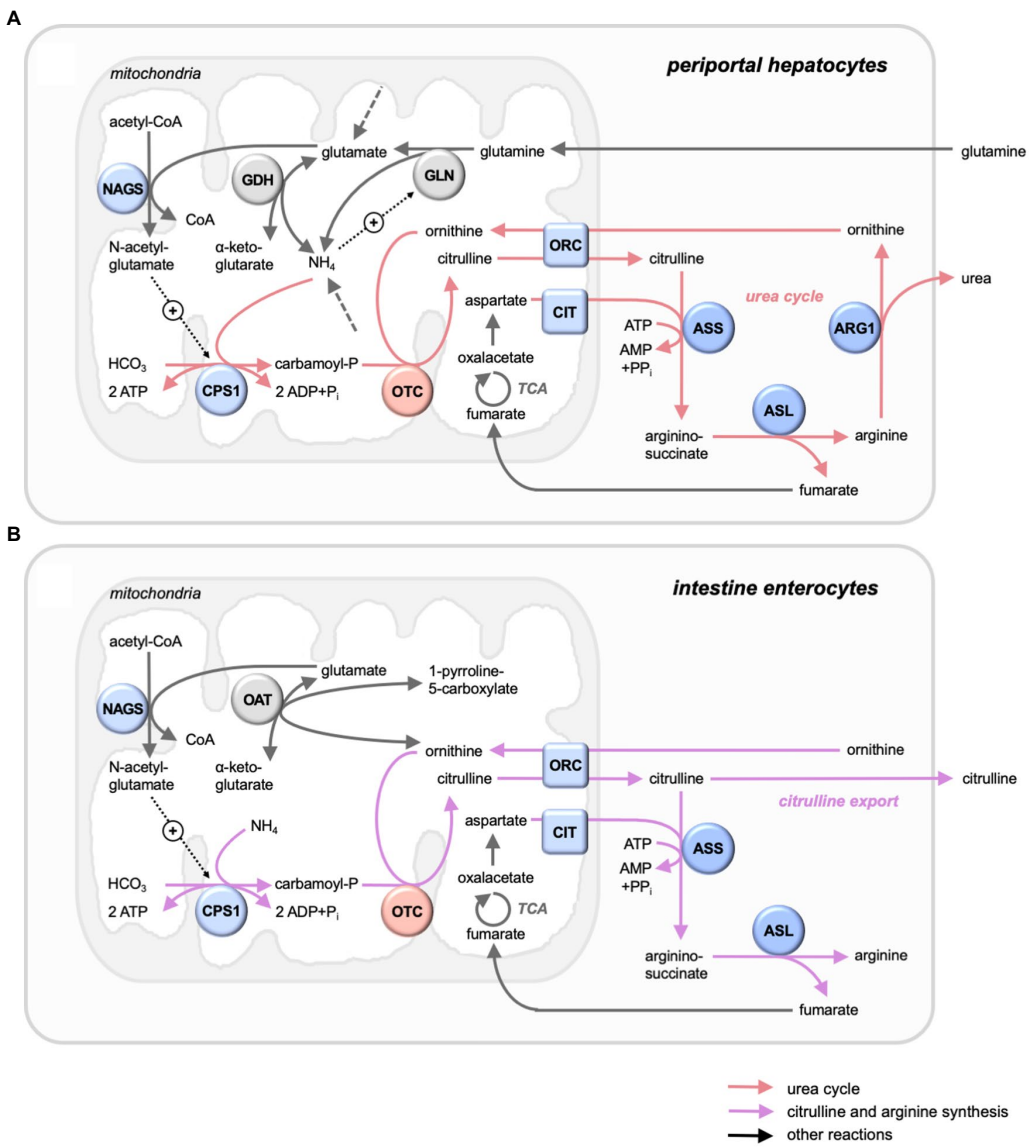
Mitochondrial OTC in its metabolic context in liver and intestine. Selected metabolic pathways with key enzymes and metabolites. **(A)** Periportal hepatocytes, with urea cycle (red arrows), running the complete urea cycle for NH_4_^+^ detoxification with all citrulline channeled into the cycle. **(B)** Intestine mucosa enterocytes, with citrulline and arginine synthesis (magenta arrows), generating mainly citrulline for export. Also shown are further reactions (grey arrows), entry points of nitrogenous compounds not detailed here (dashed grey arrows), and allosteric activations (dotted black arrows with +). Enzymes (circles) and transporters (squares) of the urea cycle (mitochondria: blue/red; cytosol: dark blue) and others (grey). The urea cycle comprises the mitochondrial enzymes carbamoyl phosphate synthase 1 (CPS1) and ornithine carbamoyltransferase (OTC), the inner membrane transporters ornithine carrier 1 (ORC, or ornithine transporter 1, ORNT1, SLC25A15) and citrin (CTR, or Ca-binding mitochondrial carrier protein Aralar2, SLC25A13), the cytosolic proteins argininosuccinate synthase (ASS), argininosuccinate lyase (ASL), and arginase 1 (ARG1), as well as generation of an allosteric activator by acetylglutamate synthase (NAGS). Glutaminolysis comprises mitochondrial glutaminase (GLN) and glutamate dehydrogenase (GDH). Ornithine and glutamate are linked by mitochondrial ornithine aminotransferase.

Anabolic OTCs catalyze the transfer a carbamoyl group from carbamoyl phosphate (CP) to the amino group of L-ornithine (ORN), yielding citrulline (CIT) and phosphate. This anabolic two-substrate reaction is involved in essential metabolic pathways such as the biosynthesis of citrulline and arginine, ammonia homeostasis, and the urea cycle in mammals (Figure 1). In eukaryotes, OTCs are localized in the mitochondrial matrix space (Takiguchi et al., 1989) where they assemble into homotrimers (or oligomers thereof) as basic catalytic unit (Shi et al., 1998). These anabolic OTCs differ from catabolic OTCs that promote the reverse reaction and are only found in lower microorganisms that use arginine as a carbon source for ATP generation. These latter OTCs are not further considered here.

In mammals, OTC is mainly expressed in liver and intestine. Hepatic OTC is an integral part of the urea cycle, which captures nitrogen in the form of toxic ammonia to generate urea for excretion (Figure 1A). The CIT generated here is entirely channeled into the urea cycle, without exchanging with the rest of the organism, and thus not involved in systemic CIT metabolism (Jourdan et al., 2008). However, significant CIT synthesis by OTC occurs in the intestine epithelial (Figure 1B), where it plays a major role in amino acid homeostasis, particularly of L-glutamine and L-arginine (Breuillard et al., 2015). Dysregulated expression of OTC in these and other tissues rewires nitrogen toward specific anabolic pathways, thus supporting growth and aggressiveness of tumors (Keshet et al., 2018) or participating in other pathologies.

Ornithine transcarbamylases have been rather well studied for several aspects, including their molecular structure, biochemical activity, and physiological functions in liver and intestinal mucosa. Much research has been motivated by OTC deficiency, a complete or partial lack of OTC that causes the most common urea cycle disorder in humans (Lichter-Konecki et al., 1993). This X-linked genetic disease presents with hyperammonemia and can further lead to neurological impairments, coma, and even death if not treated correctly. To date, options for treatment are limited, and disease management remains a challenge (Soria et al., 2019).

This review summarizes essential molecular, biochemical, and cellular data on mainly human OTC and gives an overview on the pathophysiology of OTC deficiency. Further information can be found in recent reviews on related topics, including the transcarbamylase protein family (Shi et al., 2015) and CP metabolism (Shi et al., 2018).

## OTC Genes and Proteins

The OTC gene and protein structures are well characterized in several species from bacteria to the human being. More recently, findings on OTC dysregulation in pathological states and by secondary protein modifications have sparked some new interest in OTC.

### Gene Structure, Regulation of Transcription and Translation

Most organisms carry a single, nuclear *OTC* gene. Only some invertebrates like nematodes (including *Caenorhabdidis*) and most insects lack *OTC*, with the exception of honeybee that has both anabolic and catabolic *OTCs* (Shi et al., 2015). The human *OTC* gene was isolated and characterized over 30years ago (Hata et al., 1988). It is located on the short arm of the X-chromosome (Xp21.1) and spans about 73kb (Horwich et al., 1984; Lindgren et al., 1984). The gene is composed of 10 coding exons and nine introns, with exon 3 encoding the CP-binding site and exon 9 the ORN-binding site (Kraus et al., 1985). The *OTC* gene structure is well conserved between species; the homology between human, rat, and mice varies between 75 and 92%. However, there are some species-specific differences, including the regulatory 5' flanking region (Hata et al., 1986; Veres et al., 1986; Takiguchi et al., 1987).

Translation of the mammalian *OTC* gene is controlled by two elements: a promoter at the 5' extremity of the gene and an enhancer situated 11kb further upstream of the transcription start site (Veres et al., 1986; Murakami et al., 1990; Takiguchi and Mori, 1995). These two regulatory elements provide tissue-specific regulation of mammalian *OTC* expression, with physiological OTC levels in intestinal epithelia depending on promoter activation, while those in liver depend on additional enhancer activation (Takiguchi and Mori, 1995; Luksan et al., 2010). Indeed, the *OTC* enhancer is activated by simultaneous binding of at least two liver-selective transcription factors, the hepatocyte nuclear factor 4 (HNF-4) and the CCAAT/enhancer-binding protein beta (C/EBPß; Nishiyori et al., 1994; Takiguchi and Mori, 1995). The *OTC* promoter also requires HNF-4, which is expressed in liver, but also in small intestine (Takiguchi and Mori, 1995). Further promoter activation is provided by the ubiquitous peroxisome proliferator-activated receptor gamma coactivator 1-alpha (PGC1α), a key regulator of mitochondrial metabolism (Su et al., 2018), while the COUP transcription factor 2 (COUP-TF2 or NRF2F2) is inhibitory (Kimura et al., 1993). *OTC* promoter activity may be further controlled epigenetically by hypermethylation (Delers et al., 1984), although this has not been analyzed in more detail. Mutations in the promoter and enhancer elements can also be at the origin of human OTC deficiencies (Jang et al., 2018). Less is known on factors regulating *OTC* translation. More recently, it was shown that p53, the tumor suppressor most commonly inactivated in human cancers, is repressing translation of different urea cycle enzymes, including *CPS1*, *OTC*, and *ARG1* (Lacroix et al., 2020).

Regulation of hepatic OTC expression at the transcriptional or translational level mostly depends on nutritional state and occurs coordinated with other urea cycle proteins (see also chapter iv.c). For example, high-fat diet in hamsters decreased OTC protein levels (Liao et al., 2015), while high-protein diet in mice increased *OTC* mRNA and protein (Heibel et al., 2019). The latter occurred within a generally upregulated amino acid degradation, while carbohydrate and fat metabolism were downregulated. This metabolic switch could be linked to reduce signaling by the metabolic sensor AMP-activated protein kinase (AMPK), since the AMPK inhibitor AICAR also increased OTC mRNA in human hepatocytes (Heibel et al., 2019). Despite its interest, this study has several limitations. First, both studied groups have hyperprotein intake (21.5 and 65.3% of the total energy intake; classic normal intakes are around 17%). Moreover, the authors have only evaluated protein or gene expressions that do not necessarily reflect the activity of the different enzymes.

### Protein Maturation, Targeting, and Secondary Modification

Ornithine transcarbamylase is synthesized in the cytoplasm as a precursor (38–40kDa depending on species; 39.9 kDa and 354 amino acids for human OTC). With a half-life of only 1–2min (Mori et al., 1982), this pre-protein is rapidly targeted to the mitochondria *via* a canonical N-terminal signaling peptide that drives the energy-dependent import into the mitochondrial matrix (Conboy and Rosenberg, 1981). Here, the signaling leader peptide is cleaved off by matrix proteases (Kraus et al., 1988), yielding the mature OTC protein (35–37kDa, depending on species; 36.1kDa and 321 amino acids for mature human OTC; (Pierson et al., 1977). After folding and assembling into homotrimers (in eukaryotes) or multimers [some bacteria and archaea; (Shi et al., 2015)], OTC can attach to the inner mitochondrial membrane by non-covalent interactions with phospholipids, at least in mammals (Yokota and Mori, 1986; Powers-Lee et al., 1987). In liver, OTC can represent 3–4% of the total amount of mitochondrial proteins (Clarke, 1976). The half-life of the active OTC is tissue-dependent; in rat liver, it varies between 6 and 9days (Wallace et al., 1986; Vargas et al., 1987).

**Figure 2:**
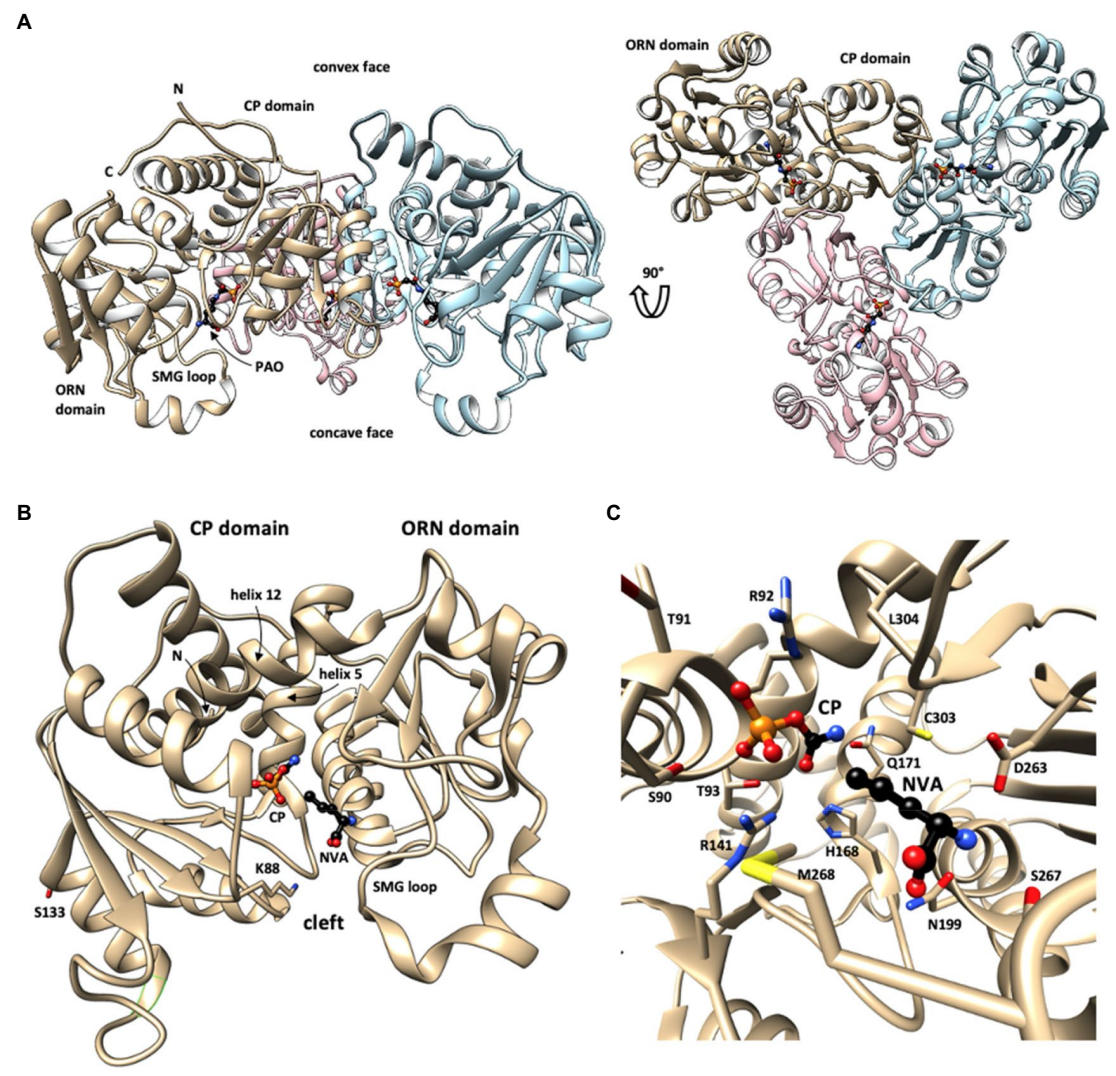
Structure of human OTC. **(A)** Overall structure of the OTC homotrimer with bound phosphonoacetyl-L-ornithine (PAO) in sideview (left) and top view (right), revealing the 3-fold rotational symmetry (every monomer in different color; one monomer labeled). This structure shows a largely open state of OTC monomers. **(B)** Fold of the closed state of monomeric OTC with N-terminal (CP) and C-terminal (ORN) domains. Long helices linking the two domains (helices 5 and 12) are labeled. Substrate-binding sites in the catalytic cleft between the two domains are identified by bound CP and L-norvaline (NVA, an ORN analogue). Selected residues involved in identified secondary modifications (acetylation at lysine 88, phosphorylation at serine 123) are shown. **(C)** Close-up showing bound substrates and involved critical OTC residues (see text). OTC structural data were taken from PDB 1OTH (A) and 1PDB C9Y **(B,C)**. The structures are given in backbone representation with individual sidechains visualized in stick representation and substrates shown as ball-and-stick models. N,C: N- and C-termini. Figure prepared with UCSF Chimera v1.11.2.

OTC can also undergo various secondary protein modifications, including many acetylations and succinylations (Figure 2B). Their physiological function is not yet entirely clear. Acetylation of K88 was shown to reduce OTC activity in response to nutrient signals (Yu et al., 2009). Inversely, deacetylation of OTC (including the K88 site) by the sirtuin Sirt3 in fasted mice increased OTC activity, while inhibition of deacetylation in Sirt3^−/−^ mice again reduced metabolite flux through the liver urea cycle and citrulline synthesis as determined in the blood of fasted mice (Hallows et al., 2011). The critical K88 is situated in the active site of OTC where it contributes to a network of hydrogen bonds that participate in the binding of the substrate CP. K88 acetylation impairs this network and reduces affinity for CP (Yu et al., 2009). Collectively, these data indicate that OTC acetylation could be a key mechanism for negatively regulating its enzymatic activity by the metabolic status of the cell, an aspect that should be examined further. There is also evidence for a phosphosite at S133 (Bian et al., 2014), but the involved kinase and its physiological function are unknown.

### Protein Structure, Active Site, and Catalysis

The molecular structure of OTC was solved for various prokaryotic and eukaryotic species, initially mainly for bacteria like *Escherichia coli* (Ha et al., 1997; Langley et al., 2000), but followed also by mammalian OTCs including four structures of human OTC with different ligands (Shi et al., 1998, 2000, 2001). These studies exploited OTC inhibitors, in particular N(5)-phosphonoacetyl-l-ornithine [PALO; Hoogenraad, 1978 and L-norvaline (Pierson et al., 1977)], to obtain high-resolution structures and define substrate interaction and catalysis. L-norvaline together with CP forms a tetrahedral transition state analogue complex, while PALO acts alone as a competitive bisubstrate analogue for both substrates. The human enzyme, like almost all anabolic OTCs, forms dish-like homotrimers with exact 3-fold symmetry and a diameter of about 100Å (Figure 2A; all numbering according to nascent human OTC). All active sites open to the concave face.

The monomer subunit has a bilobal structure. It is formed by two distinct structural domains that surround a deep cleft, linked by two long interdomain helices (h5 and h12; Figure 2B). Both structural domains have an αβα-topology, basically consisting of a central 5-stranded parallel β-sheet with α-helices on both sides (Shi et al., 2000; Diaz-Munoz and Hernandez-Munoz, 2010). Each subunit contains one active site situated at the hinge between its two domains. It involves residues in the cleft, the mobile SMG loop in the ORN domain (named after its signature peptide S267-M268-G269), and additional residues at the surface of an adjacent subunit (Shi et al., 2000; Diaz-Munoz and Hernandez-Munoz, 2010). The latter provides a rational for the obligatory requirement of trimeric OTC as the basic catalytic unit.

The CP-binding pocket is located at the bottom of the cleft involving the N-terminal CP domain, while the ORN site is at the edge of the cleft of the C-terminal ORN domain (Figures 2B,C). Occupation of the latter site partially precludes access to the CP pocket, which explains the ordered bi-bi mechanism, where CP binds first, while ORN binds second. Binding of the two substrates induces domain movements that shield the active site (Ha et al., 1997). CP binding induces a global conformational change that closes the active site cleft. Subsequent ORN binding triggers an induced fit mechanism that finally moves the SMG loop to further shield the active site and fixes it in that position by direct interactions with ORN (Figure 2B). These conformational changes were evidenced already earlier by spectroscopic methods (Reichard, 1960; Colombo and Richterich, 1968; De Gregorio et al., 1999). The CP domains also provide all the subunit contacts that form the dish-like shape of OTC trimers (Figure 2A).

Substrate recognition and binding directly or indirectly depend on various conserved OTC motifs and residues. In case of CP, they are composed of the SxRT (for the phosphate moiety; S90, T91, R92, T93) and HPxQ (for the carbamoyl moiety; H168, Q171) motifs, as well as R141, C303, L304, and Q114 from the adjacent subunit. In case of ORN, there are the DxxxSMG motif in the SMG loop (D263, S267, M268), as well as N199 and L304 (Figure 2C). In the thermodynamically favored forward reaction, OTC transfers the CP carbamoyl group to the δ amino group of the ORN side chain. As in all transcarbamylases, nucleophilic attack of the CP carbonyl carbon by the N^δ^ of ORN occurs *via* a tetrahedral transition state, which is stabilized among others by C303 and L304 (Langley et al., 2000; Shi et al., 2015; for the catalyzed reaction see also chapter iv.1).

### Structural and Functional Interactions in the Urea Cycle

Some data indicate that enzymes of the urea cycle co-localize and preferentially exchange substrates and products, a phenomenon called metabolite channeling (Srere, 1987). OTC interacts with mitochondrial carbamoyl phosphate synthetase 1 (CPS1; Holden et al., 1999; Huttlin et al., 2017), which catalyzes the first and rate-limiting step of the urea cycle by converting bicarbonate and ammonia into the OTC substrate CP in a reaction consuming 2 ATP (Figure 1). CPS1 is a matrix enzyme that co-localizes with OTC at the mitochondrial inner membrane by binding to cardiolipin and other anionic phospholipids (Powers-Lee et al., 1987; Brandt and Powers-Lee, 1991). Formation of CPS1-OTC complexes would regulate the channeling of CP into the OTC reaction that has been observed experimentally (Cohen et al., 1992). CPS1 can further interact with mitochondrial N-acetylglutamate synthase (NAGS) possibly *via* the variable segment domain (Haskins et al., 2020). NAGS has a key regulatory function in generating N-acetyl-glutamate, a mandatory positive allosteric effector of CPS (Figure 1). A more recent study confirmed that the three mitochondrial urea cycle enzymes NAGS, CPS1, and OTC can interact and form clusters at the inner mitochondrial membrane (Haskins et al., 2020) which would facilitate metabolite flux through the mitochondrial part of the urea cycle.

The second substrate of OTC, ORN, enters mitochondria in exchange with CIT *via* ornithine carriers (ORC) located in the mitochondrial inner membrane (Figure 1). Those include ORC1 (ORNT1, SLC25A15) and ORC2 (ORNT2, SLC25A2; Monné et al., 2019). Imported ORN could be directly channeled to the OTC bound at the inner mitochondrial membrane (Cohen et al., 1992). In mammalian liver, CIT exported into the cytosol is guided into the urea cycle (Figure 1A) and not available for cellular export (Jourdan et al., 2008). Indeed, tracer experiments suggested that also the three cytosolic enzymes of the urea cycle are spatially organized in a way that allows tight metabolite channeling between them (Cheung et al., 1989).

## OTC Tissue Distribution and Regulation

In human, OTC is found in several organs (Table 1); however, its activity is significant only in liver and intestine (Jones et al., 1961). In mammals, OTC is essentially hepatic since CIT synthesis is a key step in the hepatic urea cycle. In the digestive tract, where protein digestion and absorption in form of amino acids occurs, immunocytochemistry confirmed the presence of OTC in the small intestine and its absence in the stomach and the large intestine (Hamano et al., 1988). Based on mitochondrial density measurements of the same study, OTC is twice less abundant in the large intestine than in the liver. This is consistent with a gene expression study showing that *OTC* mRNA concentration in the intestinal mucosa is about half of the one found in the liver (Ryall et al., 1985). OTC activity varies along the intestine (i.e., duodenum, jejunum, ileum, colon), being more important in the proximal intestine (Raijman, 1974a). Since the proper functioning of OTC is essential to avoid hyperammonemia, it is not surprising to find OTC also in other tissues. In particular, in the brain, OTC seems to be implicated in neural modulation (Lopes-Marques et al., 2012). For example, OTC gene expression in the brain increases in subjects with Alzheimer disease (Bensemain et al., 2009; Hansmannel et al., 2009).

**Table 1:** OTC enzyme activity in tissues (From Jones et al., 1961).

Tissues	OTC enzyme activity (μmol.g^−1^.h^−1^)
Liver	4,110
Intestinal mucosa (small intestine)	100
Kidney	5
Salivary glands	12
Pancreas	2
Adrenal glands	3
Thymus	2
Lungs	2
Muscles	0
Heart	0
Brain	0
Spleen	0

**Figure 3:**
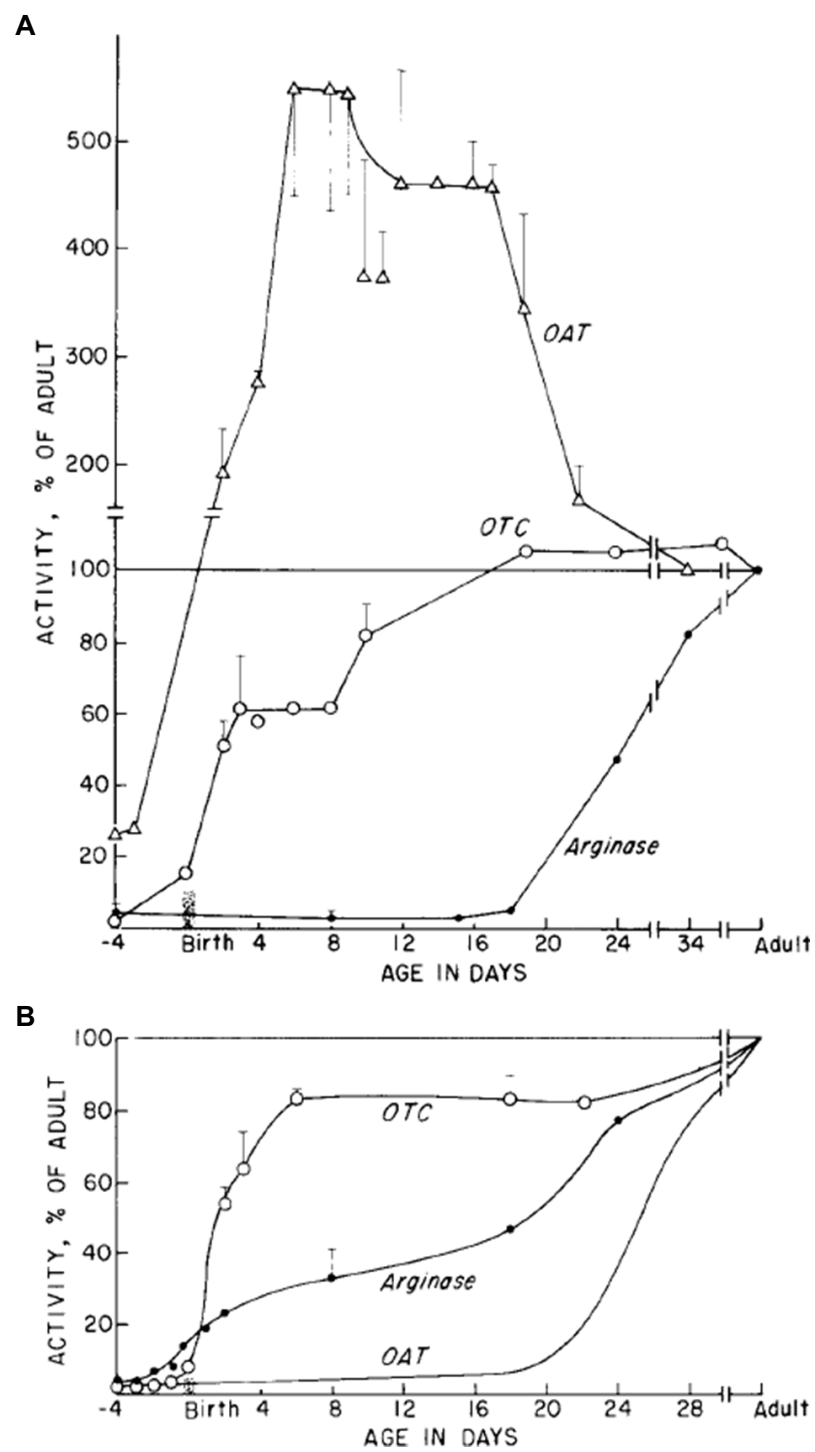
OTC, OAT, and arginase enzymes activity during development. Relative activities as percentage of activity in adult stage are shown for **(A)** small intestine and **(B)** the liver (Taken from Herzfeld and Knox, 1968).

In rats, OTC enzyme activity sets in rapidly after birth, both in the liver and in the intestine. Its accumulation is significant in the first 3weeks of life and reaches its adult value at the time of weaning (Figure 3; Herzfeld and Knox, 1968). In human, OTC starts to be expressed after 50days of gestation and its adult value is reached a few weeks before birth (Colombo and Richterich, 1968). This suggests that the enzyme could be important to sustain the metabolic needs of the rapidly developing newborn. Reichard *et al*. (Reichard, 1960, 19) have shown that the OTC enzyme activity in the intestinal mucosa corresponds to 14% of its liver activity. In the intestine, OTC activity, determined through circulating CIT levels, is a marker of enterocyte mass and function. For example, postabsorptive circulating CIT concentration is negatively correlated with remnant small bowel length in patients with short-bowel syndrome (Crenn et al., 2000) and decreased with declining enterocyte mass in patients with villous atrophy diseases (Crenn et al., 2003).

## OTC Reaction and Its Regulation

### Catalysis

Ornithine transcarbamylase is an enzyme from the carbamoyl transferase family, which means, it transfers a carbamoyl (-CO-NH_2_) functional group from the CP molecule to an amine group of the second substrate. The aspartate carbamoyltransferase (ACTase), whose second substrate is L-aspartate, and OTC, whose second substrate is ORN, are the most studied enzymes in this family (Allewell et al., 1999). Theoretically, OTC allows the production of CIT from ORN and CP and also the reverse catabolic reaction:


$$\begin{array}{l} L-Ornithine+Carbamoylphosphate↔\\ L-Citrulline+Phosphate+H+ \end{array}$$

However, thermodynamics strongly favor formation of CIT (Reichard, 1960), with a standard Gibbs free energy (ΔG°) of −63kcal/mol (Caspi et al., 2014). At 100mM ORN and 30mM CP, the apparent equilibrium constant K_eq_ is about 10^5^ (Reichard, 1957).

The reaction catalyzed by OTC occurs *via* an ordered bi-bi mechanism and follows Michaelis–Menten kinetics. The enzyme first binds CP to form a binary complex with different conformation which then allows binding of the second substrate, ORN. The specific conformation of this enzyme-substrate complex stabilizes the transition state. Here, the ORN amine group initiates a nucleophilic attach on the CP carbonyl, forming a tetrahedral intermediate. In this conformation, deprotonation of the δ-NH3^+^ group is avoided and transcarbamoylation is favored. The collapse of the transition state then eliminates P_i_ with concomitant deprotonation of the resulting CIT. Product release is also ordered, with CIT released first and P_i_ following thereafter (Marshall and Cohen, 1972; Legrain and Stalon, 1976; Wargnies et al., 1978; for structural details of catalysis see chapter ii.c).

*In vitro*, CIT synthesis is experimentally favored at temperatures of 37–38°C, a pH between 7.6 and 8.1, and substrate concentrations (CP and ORN) of approximately 0.1M. Purified hepatic OTC has a Km of 0.26mM for CP and a Km of 0.4mM for ORN at a pH of 7.7 (Lusty et al., 1979; Goldsmith et al., 1991). Variation in pH does not affect the OTC affinity constant for CP, but ORN affinity decreases with increasing pH (between pH 6 and 8; Raijman, 1974b). OTC can also use other basic amino acids like L-lysine as a second substrate (Marshall and Cohen, 1972), which, in the case of lysine, yields homocitrulline. However, affinity for lysine is much lower than for ORN, rendering this reaction negligible under physiological conditions (Ryan and Wells, 1964). Under optimal reaction conditions (37°C, pH 7.7), homocitrulline synthesis corresponds to only 0.16% of CIT synthesis (Marshall and Cohen, 1972).

### Regulation

Several parameters could influence OTC enzyme efficiency and thus affect CIT production. There are secondary modifications that regulate OTC, in particular acetylation at K88 (see chapter ii.b). However, there are also numerous allosteric and competitive effects by ions and metabolites that can affect OTC enzyme activity.

Studies on *E. coli* OTC identified Zn^2+^ ions as regulators of the enzyme. Micromolar concentrations of Zn^2+^ can act *via* two mechanisms, either as a slow, tight-binding inhibitor of the free enzyme, or as allosteric cofactor of the substrate-bound enzyme (Lee et al., 1990). In the first case, Zn^2+^ interacts with OTC in the absence of bound substrates and pH<7 to promote protein isomerization which leads to enzyme inactivation. In the second case, with CP bound, Zn^2+^ binds co-operatively to the binary complex as a reversible competitive inhibitor of ORN, without inducing enzyme isomerization.

Amino acids that are structurally related to ORN can also negatively affect OTC enzyme activity (Table 2). Inhibition by these amino acids is always competitive (Lusty et al., 1979). L-Norvaline, the non-proteinogenous, linear isomer of valine, is among the most efficient inhibitors. Its binding to the binary OTC-CP complex occurs almost as with ORN (Shi et al., 2000), and thus, inhibition is particularly strong, with a K_i_=71μM for the enzyme from rat liver (Lusty et al., 1979). Moreover, OTC can be inhibited quite effectively by L-α-aminobutyrate and L-leucine and to a lesser extent by L-isoleucine or the L-valine. Also, L-lysine is able to inhibit OTC activity, but its inhibition constant is not linear (Lusty et al., 1979). Among other metabolites, several carboxylic acids (at 10mM), nucleotides, and cofactors were tested for their effects on OTC enzyme activity. Among them, only P_i_ seems to inhibit the forward reaction, as it is also a product of the catalyzed reaction (Lusty et al., 1979). Studies on OTC in mammals have shown that the enzyme is not regulated in an allosteric way by these metabolites. Inhibitors bind at the active site, in competition with ORN, but they do not induce the conformational changes necessary for catalysis.

**Table 2:** Inhibition constant of the OTC substrate in rat liver (From Lusty et al., 1979).

Inhibitor AAs	Ki (mM)
L-Norvaline	0.071
L-α-Aminobutyrate	0.58
L-Leucine	1.20
L-Isoleucine	10.5
L-Valine	12.5
L-Histidine	40
L-Methionine	≈50
L-Glutamine	≈50
L-Lysine	NA
Pi	0.25

*In summary, these observations support the notion that OTC enzyme activity is mainly regulated by intra-mitochondria concentrations of CP and ORN*.

## Metabolic Disease

### Mutations

All the *OTC* gene mutations currently identified affect the hepatic system and more specifically the urea cycle. OTC deficiencies (OTCd) are inborn errors of the metabolism and represent approximately 50% of the urea cycle hereditary disorders leading to hyperammonemia and, without therapeutic care, to neurotoxicity (Lichter-Konecki et al., 1993; Brusilow and Horwich, 2001; Seminara et al., 2010). These deficiencies have been identified, and they are subjected to numerous publications (Tuchman, 1993; Tuchman and Plante, 1995; Tuchman et al., 2002; Yamaguchi et al., 2006). One of the most recent inventories is the one of Caldovic et al. (2015), which references 538 variants linked to hyperammonemia or OTC deficiency, based on last release of the professional database HGMD Pro (2020/08/01). A total of 538 mutations have been described in literature: 28.81% (155 variants) are loss of function mutations (frameshifts, gross deletions, gross duplications, complex mutations, and nonsense mutations), 57.06% (307 variants) are affecting the sequence of the protein (missense and inframe deletions or inframe insertions), 11.52% (62 variants) are predicted to affect consensus splice sites, 1.49% (eight variants) are affecting the promotor, 0.74% (four variants) are linked to a loss of start codon, and finally, 0.37% (two variants) are linked to a loss of STOP codons.

Caldovic et al. (2015) estimate the prevalence of these anomalies around 1/62000–1/77000. This deficiency is X-linked with recessive inheritance. The appearance of symptoms in OTCd patients is very variable, depending on the sex of the individual, but also due to X-skewed inactivation (Yorifuji et al., 1998; Musalkova et al., 2018). Heterozygous women and men with partial anomaly can report late symptoms during adulthood (McCullough et al., 2000). In these subjects, mutations can be asymptomatic or go along with symptoms with variable gravity. Most of the OTCd patients are hemizygous men and only 20% of women with genetic anomaly report symptoms (Maestri et al., 1996, 1998).

### Disease

Symptoms resulting from OTCd are linked to the toxic effects of excess ammonia. This OTCd presents phenotypic heterogeneity. The neonatal-onset type is very severe. After a free interval (a few hours or a few days), the baby suffers from hypotonia and somnolence. The most severe cases can lead until hyperammonemic coma, cerebral edema, or even death. A full deficiency often presents with severe hyperammonemic coma during the first weeks of life with an acute growth retardation leading to death (Hudak et al., 1985; McCullough et al., 2000). The late-onset type can present at any age by chronic vomiting, neurological and behavioral alterations, and clinical signs like those observed in the Reye syndrome (Yokoi et al., 1981; Mizoguchi et al., 1990; Glasgow and Middleton, 2001). It may be even diagnosed or discovered in asymptomatic patients with only biochemical abnormalities. In both types, environmental stressors (i.e., fasting, high-protein diet, pregnancy and the postpartum period, intercurrent illness, or surgery) can trigger episodes of hyperammonemic encephalopathy along with nausea, vomiting, headaches, erratic behavior, delirium, and combativeness. Many females can be affected as males because of X-skewed inactivation, with the pathogenic allele more expressed than the wild-type allele. Importantly, the neonatal cases have poorer outcome than those who present later in life. However, both forms present neurocognitive and behavioral impairment and prediction is difficult in so far as there is no correlation between the molecular defect, the ammonia concentration, and the phenotype. If the consequences of OTC deficiency in liver and its consequences on urea cycle disorders are well known, no current study has yet related major consequences of these mutations on intestinal OTC.

### Diagnosis and Treatments

Biochemical diagnosis of OTCd must be performed very quickly. A plasma amino acid chromatography is mandatory together with a urine orotic acid assay. Moreover, plasma ammonia excess is mostly combined with other biochemical alterations such as an increase in glutamine plasma level and a hypocitrullinemia (Yamaguchi et al., 2006). The high urinary excretion of orotic acid allows the distinction between OTCd patients and others with urea cycle disorders (Yamaguchi et al., 2006). Currently, the main diagnosis to confirm the OTCd is by DNA sequencing, allowing to reveal symptomatic and asymptomatic anomalies in a non-invasive way (Caldovic et al., 2015). Other techniques such as enzyme quantification in liver biopsies or orotic acid quantification in urine after protein ingestion (1g/kg) can also be used (Tuchman et al., 2002). However, these procedures are almost no longer performed because they are either too invasive (biopsy) or too dangerous (protein load).

In an emergency situation, intake of exogenous protein should be stopped and the catabolic situation should be reversed by parenteral glucose. Ammonia-scavenging drugs (sodium benzoate, sodium phenylbutyrate) are useful (Häberle, 2011; Häberle et al., 2012). In case of very high ammonia level, hemodialysis or hemofiltration is necessary. If treatment starts too late, patients have a high mortality rate due to irreversible brain edema. The current chronic therapies are based on protein diet restriction associated with medicinal therapies allowing nitrogen clearance. Usually, patients are consuming CIT and/or ARG to fight the hyperammonemia. To date, no consensus has been reached to decide which approach is the most relevant for patients. However, all these therapies do not protect the patients from hyperammonemia crisis. In the most severe cases, a liver transplant can be used as therapy (Leonard and McKiernan, 2004; Morioka et al., 2005). In a pioneering paper, Moscioni et al. (2006) demonstrated that adeno-associated virus (AAV) treatment was efficient to induce a long-term correction of ammonia metabolism and to increase life span. However, numerous liver nodules or tumors were observed in treated animals (Bell et al., 2006). Recently, teamwork focused on gene therapy as OTCd treatment by using recombinant vectors based on the adeno-associated virus serotype 8 (AAV8). This technique has been explored in Sp-fash mice, allowing to partially restore the OTC hepatic activity and to decrease the orotic acid excretion (Wang et al., 2012). Wang et al. (2017) have shown in an OTC-KO mice model that a precocious start of this therapy can limit liver alterations like fibrosis and cirrhosis induced by OTCd. A phase one clinical trial is in process to evaluate the tolerance and the efficiency of the AAV8 OTC vector (NCT02651675; Bryson et al., 2017). A similar clinical trial is also in process (Ultragenyx Pharmaceutical Inc, 2021). A second strategy is in process to evaluate the tolerance for the mRNA drug MRT5201 (NCT03767270; Prieve et al., 2018) or the drug ARCT-810 (Arcturus Therapeutics, Inc., 2021), which consists of lipid nanoparticles that contain functional *OTC* mRNA. Finally, it is important to notice that adenovirus gene therapy was associated with a tragic death in a OTC-deficient patient (18-year-old male; Raper et al., 2003). Recent pre-clinical studies also underlined that hepatic autophagy would be an important pathway for ammonia detoxification (Soria et al., 2018). Hence, in murine model of urea cycle disorders (i.e., OTC or ASL deletion), the use of Tat-Beclin-1 (an engineered cell-permeable peptide that potently and specifically induces autophagy) is able to improve survival and to alleviate hepatic injury (Soria et al., 2021).

## OTC: an Emerging Role in Chronic Disease?

In recent years, changes in the regulation and/or expression of OTC have been highlighted in various pathologies. Dysregulated expression of OTC and other urea cycle enzymes is indeed a phenomenon common to different pathologies (Barber et al., 1985; Edvardsson et al., 2003). Many tumors show deficient expression of OTC and some other urea cycle enzymes. These comprise for example subpopulations of colorectal and hepatocellular carcinoma (Alexandrou et al., 2018; He et al., 2019), glioblastoma (Khoury et al., 2015; Choy et al., 2016), and pediatric sarcomas and brain tumors (Vardon et al., 2017). Such metabolic reprogramming would also lead to higher dependency on exogenous arginine supply, which suggests that arginine deprivation therapy could be a viable therapeutic approach for these tumors (Khoury et al., 2015; Vardon et al., 2017; Alexandrou et al., 2018). Changes in the expression level of urea cycle enzymes can maximize rewiring of nitrogen toward anabolic pathways, which finally favors tumor growth. From a finalist point of view, it can be seen as a means of sparing glutamine, which is an essential substrate for the growth and development of all rapidly renewing cells (including tumor cells; Boelens et al., 2001). This includes increased synthesis of pyrimidines, resulting in nucleotide imbalance, specific mutation patterns, and worsening of patients’ outcome (Lee et al., 2018). Consistent with this model, in hepatocellular carcinoma, lower OTC expression was associated with larger tumor size and advanced grade, and OTC silencing in derived cell cultures led to increased proliferation (He et al., 2019). In contrast, in different cancer cell lines, deficiency of p53 increases transcription of *OTC*, *CPS1*, and *ARG1*, leading to higher polyamine levels and thereby promoting proliferation (Li et al., 2019).

Downregulated expression of OTC together with other urea cycle enzymes also occurs in non-alcoholic steatohepatitis, where it leads to hyperammonemia (De Chiara et al., 2018). Such observations were confirmed in patients with urea cycle disorders that exhibit higher development of liver fibrosis (Yaplito-Lee et al., 2013). Such observations were confirmed by a pre-clinical study. In a model of *OTC*-KO mice, it was observed that animals developed liver inflammation and fibrosis. However, this was totally prevented by gene therapy, using adeno-associated virus encoding a codon-optimized human *OTC* gene (Wang et al., 2017). Conversely, OTC was increased (while other urea cycle enzymes were decreased) in the liver of a mouse Huntington disease model, leading to higher blood citrulline and ammonia that could further deteriorate the patients’ health (Chiang et al., 2007). However, citrulline produced by the liver is a pool that is not exchangeable with plasma citrulline (which is only produced by intestinal OTC). Unfortunately, these aspects were not addressed by the authors.

It appears that OTC is an enzyme whose regulation is strongly disturbed in many chronic pathologies. However, to date, it is not yet possible to say whether these modifications are consequences of the pathology and whether they will have an impact on the progression of the pathology. Furthermore, it remains to be assessed whether altered OTC expression in the gut could contribute to the etiology of chronic diseases through changes in citrulline production. Several issues can be raised in that context. First, it would be interesting to explore the extent to which altered intestinal citrulline production may contribute to the etiology of liver diseases. A similar question could be asked with respect to type 2 diabetes. In that context, plasma citrulline concentration was found to be 35% higher in subjects with chronic hyperglycemia (HbA(1c) >6.0%) compared to subjects with a normal HbA(1c; ≤6.0%). Nevertheless, L-citrulline supplementation has been shown to increase plasma nitric oxide levels and reduce arginase activity in patients with type 2 diabetes (Shatanawi et al., 2020), raising the question of the importance of citrulline availability in this condition. Finally, it would be important to better decipher the interplay between gut citrulline production and gut microbiota composition and metabolism (Kao et al., 2015), which has been implicated in the etiology of chronic diseases, particularly liver diseases (Sharpton et al., 2019) and type 2 diabetes (Zhuang et al., 2019).

## Author Contributions

MC wrote the core of the first draft. All authors contributed to write, and improve the manuscript. CM supervised the redaction and the management of the manuscript.

## Conflict of Interest

The authors declare that the research was conducted in the absence of any commercial or financial relationships that could be construed as a potential conflict of interest.

## Publisher’s Note

All claims expressed in this article are solely those of the authors and do not necessarily represent those of their affiliated organizations, or those of the publisher, the editors and the reviewers. Any product that may be evaluated in this article, or claim that may be made by its manufacturer, is not guaranteed or endorsed by the publisher.
